# A Conserved Mitochondrial ATP-binding Cassette Transporter Exports Glutathione Polysulfide for Cytosolic Metal Cofactor Assembly*[Fn FN3]^♦^

**DOI:** 10.1074/jbc.M114.553438

**Published:** 2014-07-08

**Authors:** Theresia A. Schaedler, Jeremy D. Thornton, Inga Kruse, Markus Schwarzländer, Andreas J. Meyer, Hendrik W. van Veen, Janneke Balk

**Affiliations:** From the ‡ John Innes Centre, Norwich Research Park, Norwich NR4 7UH, United Kingdom,; the § Department of Plant Sciences, University of Cambridge, Cambridge CB2 3EA, United Kingdom,; the ‖ Institute of Crop Science and Resource Conservation, University of Bonn, 53113 Bonn, Germany,; the ** Department of Pharmacology, University of Cambridge, Cambridge CB2 1PD, United Kingdom, and; the ¶ School of Biological Sciences, University of East Anglia, Norwich NR4 7TJ, United Kingdom

**Keywords:** ABC Transporter, Arabidopsis, Iron-Sulfur Protein, Mitochondria, Yeast, Glutathione, Transportomics

## Abstract

An ATP-binding cassette transporter located in the inner mitochondrial membrane is involved in iron-sulfur cluster and molybdenum cofactor assembly in the cytosol, but the transported substrate is unknown. ATM3 (ABCB25) from *Arabidopsis thaliana* and its functional orthologue Atm1 from *Saccharomyces cerevisiae* were expressed in *Lactococcus lactis* and studied in inside-out membrane vesicles and in purified form. Both proteins selectively transported glutathione disulfide (GSSG) but not reduced glutathione in agreement with a 3-fold stimulation of ATPase activity by GSSG. By contrast, Fe^2+^ alone or in combination with glutathione did not stimulate ATPase activity. *Arabidopsis atm3* mutants were hypersensitive to an inhibitor of glutathione biosynthesis and accumulated GSSG in the mitochondria. The growth phenotype of *atm3-1* was strongly enhanced by depletion of the mitochondrion-localized, GSH-dependent persulfide oxygenase ETHE1, suggesting that the physiological substrate of ATM3 contains persulfide in addition to glutathione. Consistent with this idea, a transportomics approach using mass spectrometry showed that glutathione trisulfide (GS-S-SG) was transported by Atm1. We propose that mitochondria export glutathione polysulfide, containing glutathione and persulfide, for iron-sulfur cluster assembly in the cytosol.

## Teaser

**Background:** ABC transporters of mitochondria (ATM) are required for formation of cytosolic iron-sulfur clusters and molybdenum cofactor.

**Results:** *Arabidopsis* ATM3 and yeast Atm1 transport radiolabeled glutathione disulfide (GSSG). Transport of glutathione trisulfide (GS-S-SG) was demonstrated by mass spectrometry.

**Conclusion:** A mitochondrial transporter exports glutathione polysulfide.

**Significance:** Identification of substrate(s) of ATMs defines their role in metal cofactor assembly and iron homeostasis.

## Introduction

Iron-sulfur (Fe-S)^7^ proteins perform essential functions in respiration, photosynthesis, DNA metabolism, and many other processes in different compartments of the eukaryotic cell. Mitochondria and chloroplasts harbor autonomous pathways for the assembly of Fe-S clusters (1, 2). The biogenesis of Fe-S proteins in the cytosol and nucleus requires a separate set of five to six assembly proteins but also depends on mitochondria (3, 4). An ATP-binding cassette transporter of the mitochondria (ATM) has been identified in yeast, plants, and mammals, which is required for cytosolic and nuclear Fe-S cluster assembly (5–7). It is therefore likely that the mitochondria provide a compound that is exported by the ATM transporter, but this molecule has not been identified.

The assembly of Fe-S clusters starts with the extraction of sulfur from cysteine catalyzed by cysteine desulfurase. The sulfur is bound to the enzyme in the form of persulfide, also called sulfane sulfur, with an oxidation state of 0 (RS-S^0^H). The enzyme-bound persulfide is then transferred to a scaffold protein where it is combined with iron (4). Mitochondrial localization of the cysteine desulfurase activity appears to be critical for Fe-S cluster assembly in the cytosol and nucleus in yeast and plants (3, 8), despite the occurrence of extramitochondrial cysteine desulfurases (9, 10).

The ATM proteins belong to the ABCB subfamily of ABC proteins and are half-transporters that dimerize for function. Recent crystal structures of yeast Atm1 (11) and a bacterial homologue (12) revealed their typical architecture, with two membrane domains that provide the pathway for substrate transport and two nucleotide-binding domains (NBDs) that couple the energy provided by ATP binding and hydrolysis to the translocation of substrates across the membrane. Atm1 in yeast is localized in the inner membrane of the mitochondria with the NBDs facing the matrix (13). The yeast, plant, and mammalian ATMs are functional orthologues, because *ATM3* (*ABCB25*) from the model plant *Arabidopsis* and *ABCB7* from human can complement the phenotypes of a yeast Δ*atm1* mutant (14–17).

It was initially suggested that ATMs transport an Fe-S cluster intermediate, because iron accumulates to high levels in mitochondria in yeast *atm1* mutants (18). However, recent studies have suggested that increased iron uptake is the result of disrupted iron homeostasis in the cell (19). Moreover, mitochondrial iron accumulation was not found in *Arabidopsis atm3* mutants (6) or in mouse hepatocytes depleted of ABCB7 (7). The plant *ATM3* gene has also been implicated in heavy metal resistance (20) and in molybdenum cofactor (Moco) biosynthesis (21). The assembly of Moco requires the precursor cyclic pyranopterin monophosphate synthesized in the mitochondria and two sulfur atoms of which the origin is uncertain. Therefore, ATM3 either exhibits a broad substrate specificity for different molecules or it transports a molecule shared by cytosolic Fe-S cluster assembly, Moco biosynthesis, and heavy metal detoxification.

The tripeptide glutathione has previously been implicated in the function of yeast Atm1 (22, 23). Moreover, reduced glutathione (GSH) was associated with yeast and bacterial Atm1 in the crystal structures (11, 12), whereas bacterial Atm1 also bound oxidized glutathione (glutathione disulfide, GSSG) (12). However, transport of glutathione or glutathione conjugates has not yet been shown for the mitochondrial ATM transporters.

Using our previously identified allelic series of *Arabidopsis atm3* mutants (6), we have investigated whether glutathione plays a role in the function of ATM3 in plants. In addition, various putative substrates were tested for their capacity to stimulate ATPase activity of purified *Arabidopsis* ATM3 and yeast Atm1. We found that both proteins can transport GSSG but not GSH. Further *in vitro* and genetic interaction studies provide evidence for transport of persulfide in the form of glutathione trisulfide (GS-S^0^-SG) as a physiological substrate.

## EXPERIMENTAL PROCEDURES

### 

#### 

##### Plant Materials and Growth

*Arabidopsis thaliana* ecotype Columbia (Col-0) was used as wild type in all our studies. The *atm3* mutant alleles (gene identifier *AT5G58270*) and *atm1-1* (*AT4G28630*) have been described previously (6). The *oastlC* (*AT3G59760*) knock-out mutant is described in Ref. 24, and the *ethe1-1* (*AT1G53580*) knockdown mutant in Ref. 25. Plants were germinated on half-strength Murashige and Skoog medium with 0.8% (w/v) agar and transplanted to compost after 2 weeks. Buthionine sulfoximine (BSO) and glutathione were added after autoclaving the medium. BSO was either added as pure l-BSO or the racemic mix of dl-BSO (Sigma). Plants were grown in a controlled environment at 20 °C, 65% humidity in a 16-h light/8-h dark cycle with a photon flux density of 100–120 μmol m^−2^ s^−1^.

##### Glutathione Levels and roGFP Analysis

Mitochondria were isolated from cell culture or hydroponic seedlings as described previously (6). Thiols were extracted with sulfosalicylic acid and quantified with dithionitrobenzoic acid (Ellman's reagent) in a cyclic assay using glutathione reductase (18). Alternatively, mitochondria were extracted with hydrochloric acid and labeled with monobromobimane for HPLC analysis of GSH (24). Quantitative *in vivo* imaging of the cytosolic GRX1-roGFP2 (26) and mitochondrial roGFP2-GRX1 sensor constructs (27) in 7–8-day-old seedlings was performed using a Zeiss LSM780 confocal microscope (Carl Zeiss MicroImaging GmbH, Goettingen, Germany). Image collection and ratiometric analysis were essentially as described previously (28, 29).

##### Bacterial Strains, Plasmids, and Growth Conditions

*Lactococcus lactis* strain NZ9000 Δ*lmrA* Δ*lmrCD* (30) was grown at 30 °C in M17 broth (Oxoid) supplemented with 0.5% (w/v) glucose and appropriate antibiotics for maintenance of plasmids. Cells were transformed with empty expression vector pNZ8048 (31), pNZ8048 encoding C-terminally His_6_-tagged Atm1 or Atm1 ΔK475 (this study), empty vector pERL (32), or pERL encoding C-terminally His_10_-tagged ATM3 or ATM3 E641Q (this study), downstream of a nisin A-inducible promoter. Medium was inoculated with a 1:50 dilution of overnight culture, and cells were grown to an OD_660_ of 0.5–0.6. Expression of ATM proteins was induced for 1.5 h at 30 °C in the presence of 0.1% (v/v) of nisin A-containing supernatant of the nisin-producing strain *L. lactis* NZ9700 (31).

##### Construction of ATM Mutants

The first 58 codons of the *ATM1* gene from *Saccharomyces cerevisiae* were removed, and a C-terminal His_6_ tag and XbaI site were introduced by PCR using primer Sc_tr and Sc_Rev_His (for all primer sequences see supplemental Table S1). The PCR product was cloned into pJET vector (Fermentas). Site-directed mutagenesis was performed using the QuikChange lightning kit (Stratagene) to introduce the ΔK475 mutation with primers Atm1_DK_For and Atm1_DK_Rev. Wild-type and mutant *ATM1* were reamplified by PCR and cloned into pNZ8048 to generate pNZ_Atm1 and pNZ_Atm1ΔK475.

ATM3 expression constructs were generated using the backbone exchange method (32). N-terminally truncated versions of ATM3 were amplified using ATM3_FX_Rev and ATM3_FX30_For, ATM3_FX60_For, or ATM3_FX97_For and cloned into vector pREX containing a C-terminal His_10_ tag as described (32, 33). Site-directed mutagenesis was performed using primers ATM3_EQ_For and ATM3_EQ_Rev.

##### Preparation of Inside-out Membrane Vesicles and Protein Purification

Inside-out membrane vesicles were prepared from lactococcal cells by passage through a Basic Z 0.75-kW Benchtop Cell Disruptor (Constant Systems) at 20,000 p.s.i. as described previously (34). Protein concentration was determined using the DC assay kit (Bio-Rad), and expression of the proteins was confirmed by protein blot analysis with antibodies against the His tag, ATM3, or Atm1.

Inside-out membrane vesicles (30 mg of total protein) were solubilized in 7.5 ml of Buffer A (50 mm HEPES-KOH buffer, pH 8.0, 100 mm NaCl, 10% (v/v) glycerol) containing 1.0% (w/v) β-d-dodecyl maltoside (DDM, Melford) or 1.0% (w/v) laurylmaltoside neopentylglycol (LMNPG, Affymetrix). The solubilization mixture was incubated on a rotating wheel for 2 h at 4 °C. Unsolubilized particles were removed by centrifugation at 125,000 × *g* for 30 min at 4 °C, and the supernatant was transferred to 400 μl of nickel-nitrilotriacetic acid (Ni-NTA)-agarose suspension (Qiagen) pre-equilibrated with Buffer A containing 0.1% (w/v) DDM or 0.05% (w/v) LMNPG and 30 mm imidazole. The mixture was incubated for a further 2 h and subsequently transferred to a Bio-spin column (Bio-Rad). The resin was washed with 5 column volumes of equilibration buffer and with 6 volumes of Buffer B (50 mm HEPES-KOH, pH 7.0, 100 mm NaCl, 10% (v/v) glycerol, 30 mm imidazole, 0.1% (w/v) DDM, or 0.05% (w/v) LMNPG). The protein was eluted in elution buffer (Buffer B, but containing 5% (v/v) glycerol and 250 mm imidazole). Protein concentrations were determined using the Micro-BCA assay kit (Pierce).

##### ATPase Measurements

ATPase activity of purified ATM3 and Atm1 was determined using the malachite green colorimetric assay. Briefly, 5 μg of purified protein was added to 0.1 m HEPES-KOH, pH 7.0, supplemented with 5 mm MgCl_2_ and 5 mm Na-ATP. The samples were incubated at 30 °C for 5 min, and subsequently 150 μl of malachite green solution was added (0.525 g of ammonium molybdate·7H_2_O, 17 mg of malachite green, 12.5 ml of 4 n hydrochloric acid, and MilliQ water to 50 ml), which had been activated with 0.1% Triton X-100. The absorbance was measured at a wavelength of 640 nm. Background levels of P_i_ in the elution buffer were measured and subtracted. The NADH-coupled assay was performed as described previously (35). Where indicated, compounds were added to the assay mixture, at a final concentration of 3.3 mm unless otherwise stated, to determine their ability to stimulate ATPase activity. The GSSG polysulfide mixture was prepared as described (36). Glutathione persulfide (GSSH) was produced by mixing GSSG and Na_2_S in a 1:4 ratio with subsequent incubation at 30 °C for 15 min.

##### Substrate Transport in Inside-out Membrane Vesicles

Inside-out membrane vesicles with expressed ATM proteins were prepared as described above. The transport reaction contained 1 mg/ml membrane vesicles, 5 mm ATP, 5 mm MgCl_2_, and an ATP regeneration system consisting of 0.1 mg/ml creatine kinase and 5 mm phosphocreatine (both from Roche Applied Science) in 0.25 ml of 0.1 m KP_i_, pH 7.0. [^35^S]GSH (PerkinElmer Life Sciences; 944 Ci/mmol) was mixed with nonlabeled GSH to obtain a final concentration of 250 μm [^35^S]GSH (0.95 Ci/mmol). [^35^S]GSSG was prepared from [^35^S]GSH as described in Ref. 37. In brief, dithiothreitol was removed by solvent extraction with ethyl acetate, and GSH was oxidized to GSSG by addition of 1% (v/v) H_2_O_2_. The purity of [^35^S]GSH and [^35^S]GSSG was analyzed by thin liquid chromatography on Silica 60 F254 nm plates (Merck) in 16:3:5 isopropyl alcohol/H_2_O/acetic acid. Plates were dried and exposed to Biomax MR film (Eastman Kodak) at −80 °C for 3 days.

The transport reaction was incubated for the indicated time and then filtered over nitrocellulose filters (Whatman; 0.45 μm) by rapid vacuum filtration. The filters were washed twice with 5 ml of ice-cold 0.1 m KP_i_, pH 7.0, to decrease background binding of free ^35^S-labeled compounds. Radioactivity retained on the filters was determined by liquid scintillation counting in Ultima Gold XR (PerkinElmer Life Sciences).

Transport reactions for GS-S-SG were carried out as for the radiolabeled substrate, except that the membrane vesicles were mixed with GSSG/GS-S-SG mixture containing 500 μm GSSG and 100 μm GS-S-SG as quantified by LC-MS/MS. After incubation for 30 min, samples were filtered over MultiScreen HTS+ plates (Millipore), washed with KP_i_, and eluted in 70% (v/v) acetonitrile. Samples were diluted to 50% (v/v) acetonitrile, and 15 μl was applied to a Luna 3-μm NH_2_ column (100 × 2 mm, Phenomenex) attached to a Thermo-Finnigan Surveyor HPLC system. GSSG and GS-S-SG were separated in Hilic mode using a 90 to 10% gradient of acetonitrile in 20 mm ammonium acetate, pH 9.45, over 15 min. Eluted peaks were resolved on a LCQ DECA XPplus MS equipped with an electrospray ionization source. This was operated in negative mode and set to trap ions of *m/z* 611.1 (GSSG) and *m/z* 643.1 (GS-S-SG) to detect characteristic fragments. Ion intensities were quantified by peak integration using XCalibur software (ThermoFisher Scientific) and normalized to standards in the same experiment.

## RESULTS

### 

#### 

##### Arabidopsis atm3 Mutants Are Hypersensitive to an Inhibitor of Glutathione Biosynthesis

**FIGURE 1.**
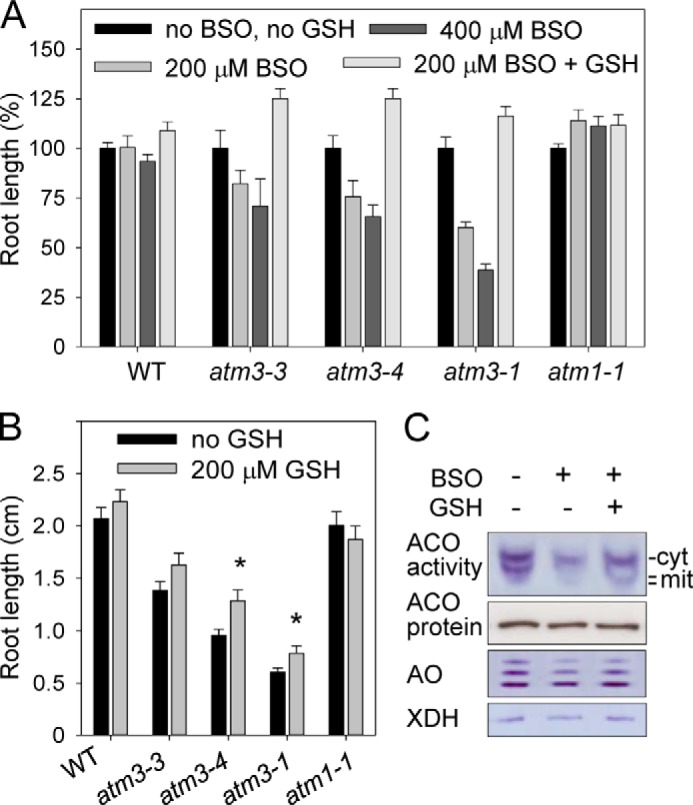
***Arabidopsis atm3* mutants are sensitive to GSH depletion.** *A,* relative root length after 8 days in wild-type (*WT*) and *atm3* mutant seedlings without any additions or with BSO (200 and 400 μm racemic dl-buthionine-(*SR*)-sulfoximine) and GSH (reduced glutathione, 200 μm) as indicated. A knock-out allele of the paralogue *ATM1* served as a control. Values represent the mean ± S.E. (*n* >25). *B,* absolute root length (in centimeters) in seedlings without (*black bars*) and with 200 μm GSH (*gray bars*). Values represent the mean ± S.E. for two independent experiments and *n* >25. *, *p* < 0.05 (Student's *t* test). *C,* activities of Fe-S enzymes in wild-type seedlings treated with BSO (400 μm l-buthionine sulfoximine) and 200 μm GSH. Seedlings were grown as in *A*. Cell extracts were separated under native conditions, and stained for aconitase activities (*ACO*, 50 μg of protein per lane), aldehyde oxidases (*AO*, 50 μg of protein per lane), and xanthine dehydrogenase (*XDH*, 30 μg of protein per lane). *cyt*, cytosolic isoform; *mit*, mitochondrial isoforms. Aldehyde oxidases and xanthine dehydrogenase are cytosolic enzymes. The same samples were subsequently analyzed for aconitase protein levels by immunoblotting under denaturing conditions, in which the isoforms are not separated and the signal represents total aconitase protein.

*Arabidopsis atm3-1* seedlings were previously shown to accumulate 2-fold more non-protein thiols such as glutathione, whereas transcript levels of the *GSH1* gene were elevated (20). To further investigate a possible interaction between glutathione and ATM3 function in plants, wild-type and *atm3* seedlings were germinated on medium with low concentrations of BSO, a specific inhibitor of glutamate-cysteine ligase (EC 6.3.2.2), which is encoded by *GSH1* and mediates the first step of glutathione synthesis (38). We tested three *atm3* mutant alleles as follows: the weak *atm3-3* allele that has an R612K substitution in the X-loop of the NBD; the intermediate *atm3-4* allele that has less than 10% expression of *ATM3* due to a 39-nucleotide deletion in the promoter; and the strong *atm3-1* allele that lacks the NBD (6). Concentrations of 200 or 400 μm BSO in the growth medium did not affect root growth of wild-type seedlings (Fig. 1*A*). By contrast, a significant decrease in relative root growth was seen in all three *atm3* mutant lines, with up to 60% inhibition in the stronger *atm3-1* allele exposed to 400 μm BSO. The degree of growth inhibition correlated with the severity of other phenotypic parameters in the *atm3* mutants (6) and could be reversed by addition of GSH to the growth medium. GSH alone had no major effect on root growth; however, a slight increase in root length was observed in the *atm3-3* and *atm3-4* mutant alleles (Fig. 1*B*). Thus, depletion of glutathione aggravates the slow growth phenotype of *atm3* mutants. The functional interaction between glutathione and the *Arabidopsis* ATM3 transporter echoes the findings in yeast, in which genetic depletion of both GSH and Atm1 is lethal (22).

Next, we investigated whether glutathione depletion had an effect on the activity of Fe-S enzymes as an indirect measure of Fe-S cluster assembly (5). For this purpose, wild-type seedlings were grown in the presence of 400 μm BSO. Cell extracts were separated by native gel electrophoresis, and activities of aconitases, aldehyde oxidases, and xanthine dehydrogenase were assayed by in-gel staining. Inhibition of glutathione biosynthesis by BSO resulted in strongly decreased aconitase activities for both the cytosolic and mitochondrial isoforms of wild-type seedlings, which was restored by the addition of GSH to the medium (Fig. 1*C*). However, the cytosolic aldehyde oxidase and xanthine dehydrogenase activities were hardly affected by GSH depletion. It is possible that the Fe_2_S_2_ clusters of the latter enzymes are more stable than the Fe_4_S_4_ cluster of aconitase.

##### Glutathione Redox State Is Shifted toward Oxidation in Mitochondria of atm3 Mutants

**FIGURE 2.**
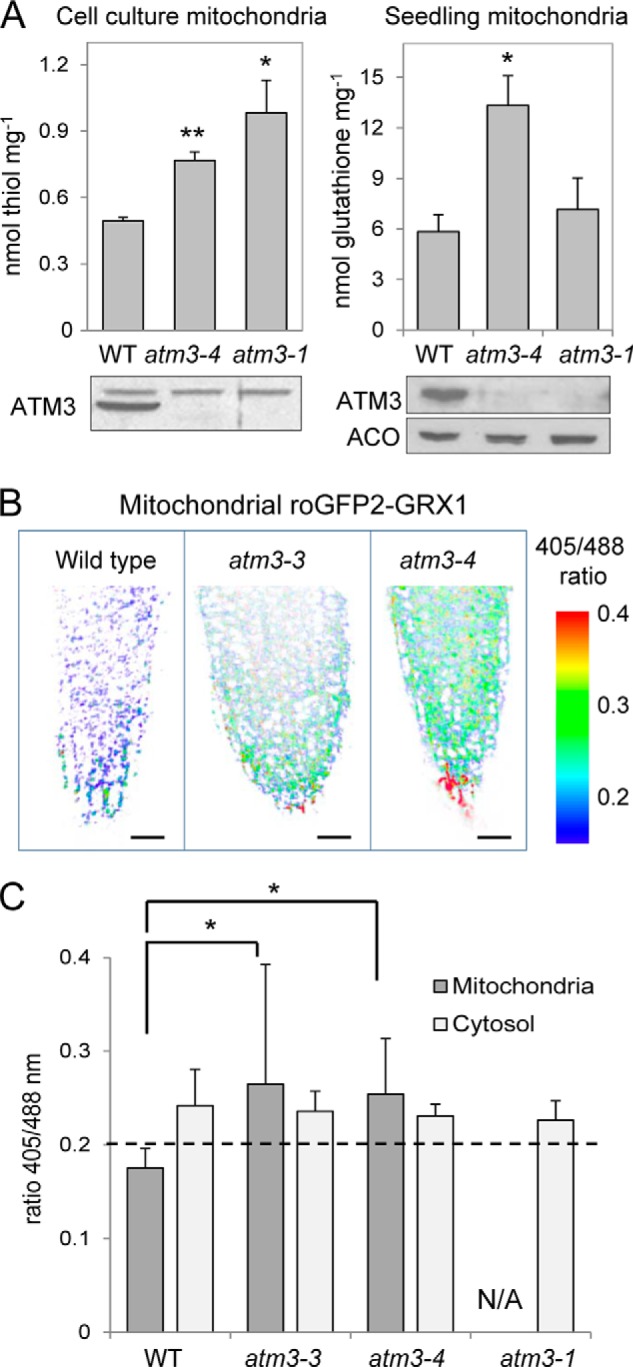
**Glutathione levels and redox status of the mitochondrial and cytosolic glutathione pools in *Arabidopsis atm3* mutants.** *A,* analysis of non-protein thiols or total glutathione levels (GSH + GSSG) in isolated mitochondria from wild-type and *atm3* mutants, from cell culture (*left panel*), or from hydroponic seedlings (*right panel*). Values are the mean ± S.E. of ≥3 mitochondrial preparations. *, *p* < 0.05; **, *p* < 0.01 (Student's *t* test). Immunoblotting confirmed the absence of ATM3 protein in mitochondria from the mutant lines. *B,* false-color images of root tips representing the readout of the ratiometric roGFP2-GRX1 sensor expressed in the mitochondria in wild-type (*WT*), *atm3-3,* and *atm3-4*. The *red* colored cells in the root cap are undergoing cell death. Note that the ratio range shown does not represent the full dynamic sensor range but has been chosen to match the ratio range of the samples. *Scale bar,* 20 μm. *C,* ratios of the emission intensities upon excitation at 405 and 488 nm in images of roGFP2-GRX1 expressed in mitochondria and cytosol in wild-type (*WT*) and *atm3* lines. The ratio provides a specific readout for the redox state of the glutathione pool and increases with oxidation, *i.e.* increased GSSG levels. The *dashed line* indicates the ratio of the fully reduced sensor (treatment with 10 mm dithiothreitol). The ratio of fully oxidized sensor was 1.27 for the cytosolic and 0.95 for the mitochondrial roGFP2 sensor construct, respectively. The values represent the mean ratio ± S.D. of 10–15 individual root tips. *, *p* < 0.05 (one-way ANOVA followed by post hoc Tukey's test; comparison of *atm3* mutants to WT). *N/A,* not available as we could not obtain *atm3-1* expressing the mitochondrial roGFP2-GRX1 construct.

The greater sensitivity of *atm3* mutants to BSO could be caused by the following: (i) lower steady-state levels of glutathione; (ii) altered distribution of glutathione in the cell; or (iii) a more oxidized glutathione pool, *i.e.* an increased GSSG to GSH ratio as seen in total cell extracts of the yeast Δ*atm1* mutant (18). A previous report on the *Arabidopsis atm3-1* mutant (20) showed that non-protein thiols such as glutathione were increased 2-fold in cell extracts, so therefore we rejected point i. To analyze whether the cellular distribution of glutathione is altered when ATM3 is not functional (point ii), we measured the glutathione content in mitochondria from wild-type and *atm3* mutants. Intact and >90% pure mitochondria were isolated from cell cultures or hydroponic seedlings, extracted under acid conditions and analyzed by the cyclic dithionitrobenzoate assay for non-protein thiols (known to be mostly glutathione) or by bromobimane derivatization and HPLC. We found that the total glutathione levels (GSH + GSSG) were significantly increased by up to 2-fold in *atm3* mitochondria (Fig. 2*A*). However, compared with the overall 2-fold increase in the cell (20), we conclude that glutathione does not specifically accumulate in the mitochondria of *atm3* mutants.

To investigate the redox state of the glutathione pool on the mitochondrial and cytosolic side of the ATM3 transporter (see point iii above), we used the redox-sensitive GFP2 (roGFP2) fused to glutaredoxin1 (GRX1). This approach reflects the *in vivo* situation and is therefore superior to cell-disruptive methods, because the glutathione pool tends to become oxidized during the isolation procedure for mitochondria. The roGFP2 sensor was targeted to the mitochondrial matrix and cytosol, respectively (27, 39). Stable expression of the roGFP2 sensors in each cell compartment was confirmed by fluorescence microscopy in wild-type, *atm3-3,* and *atm3-4* mutants (data not shown). However, for the strong *atm3-1* mutant allele, we could only obtain expression of the sensor in the cytosol but not in the mitochondria. The emission ratio upon excitation of the sensor with 405 and 488 nm provides a readout of the redox potential of the surrounding GSSG/GSH buffer and ranged from ∼0.2 (fully reduced) to 1–1.2 (fully oxidized). We found that the 405:488 ratio value of roGFP2 was significantly increased in images of the mitochondrial sensor in *atm3-3* and *atm3-4* mutants compared with wild type (Fig. 2, *B* and *C*). This observation was consistently made in two separate experiments and in different tissue types as follows: root tip, root elongation zone, and cotelydon epidermis. By contrast, the 405:488 ratio values for the cytosolic roGFP sensor were similar in *atm3* mutants and wild type.

The glutathione redox potential shifts toward oxidation either by a decrease in total glutathione levels or an increase in the GSSG/GSH ratio. Both lead to an increase in the roGFP ratio value (39). In mitochondria from *atm3* mutants, glutathione levels are higher than in wild type (Fig. 2*A*), and when glutathione was depleted using the inhibitor BSO, the 405:488 ratio of mitochondrial roGFP increased to a similar extent in wild-type and *atm3* mutants (data not shown). Therefore, an increased 405:488 ratio of the roGFP sensor indicates that the mitochondrial matrix of *atm3* mutants contains relatively more GSSG than in wild-type mitochondria. These data also suggest that if glutathione is a direct substrate of ATM3 it is preferentially transported in the oxidized disulfide form.

##### Expression and Purification of Arabidopsis ATM3 and Yeast Atm1 Using L. lactis

For functional characterization of the substrate specificity of ATM3, we chose the Gram-positive bacterium *L. lactis* as an expression system (40, 41). Yeast Atm1 was also included in our study for comparison. Protein expression in lactococcal cells has several advantages such as tight regulation of expression by the nisin-inducible promotor, low proteolytic activity, and a low rate of protein misfolding. Additionally, the *L. lactis* strain used in this study, NZ9000, cannot produce glutathione (42).

To express ATM3 and Atm1, we truncated the putative N-terminal mitochondrial targeting sequence (MTS), as this sequence is known to cause instability of the protein in bacterial expression systems. For ATM3, prediction programs (MitoProt, TargetP, and SignalProt) were inconclusive as to the length of the MTS. We therefore generated three constructs in which the first 30, 60, or 97 amino acids were deleted. All three constructs were expressed, but the Δ60 construct was most active (data not shown) and is used in this study. For Atm1, a targeting sequence of 26 residues was predicted by MitoProt and TargetP, whereas alignment with bacterial AtmA homologues suggested that the first 58 residues comprise the MTS. By contrast, 93 ± 2 amino acids were cleaved off the N terminus when Atm1 was expressed in *Escherichia coli* (23). As a consensus, we decided to remove the first 58 amino acids, which yielded stable and active Atm1. A C-terminal histidine tag was introduced in both constructs to enable purification by Ni-NTA affinity chromatography.

**FIGURE 3.**
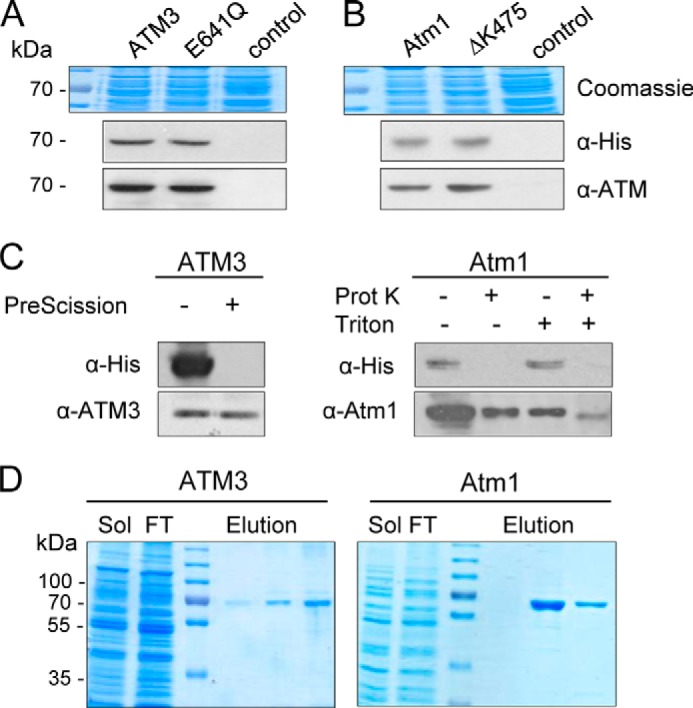
**Expression and purification of ATMs.** *Arabidopsis* ATM3 and ATM3 E641Q (*A*), and yeast Atm1 and Atm1 ΔK475 (*B*) were expressed from plasmids in *L. lactis*. Total membrane protein (24 μg) was analyzed by immunoblotting for expression of the transporter proteins using anti-His or specific antibodies. Coomassie staining confirmed equal loading. *Control*, membranes from cells carrying an empty plasmid. *C,* orientation of ATMs in inside-out membrane vesicles. Samples (24 μg of protein) were treated as indicated and then subjected to immunoblotting with anti-His or anti-ATM antibodies. ATM3 has a C-terminal His_10_ tag with a cleavage site specific for PreScission protease. Atm1 has a C-terminal His_6_ tag without a cleavage site; therefore, the nonspecific protease K (*Prot K*) was used. In both cases, the His tag is accessible to the protease, indicating that the C-terminal NBD is oriented to the outside of the membrane vesicles. Addition of the detergent Triton X-100 renders Atm1 accessible to protease K. *D,* ATM3 and Atm1 were purified to homogeneity using Ni-NTA affinity chromatography. *Sol,* solubilized membranes; *FT,* flow-through.

We also generated ATM3 and Atm1 mutant proteins with an impaired ability to hydrolyze ATP (43). Deletion of the catalytic lysine in the Walker A motif, which is often used for this purpose, destabilized the ATM3 protein; therefore, we generated E641Q adjacent to the Walker B motif. For yeast Atm1, deletion of Lys-475 in the Walker A motif did not affect its stability. Equal expression of wild-type and mutant proteins in lactococcal membranes was confirmed by protein blot analysis (Fig. 3, *A* and *B*).

To assess the orientation of ATM3 and Atm1 in the lactococcal plasma membrane, we tested the accessibility of the histidine tag at the C terminus of both proteins in well defined inside-out membrane vesicles using a membrane-impermeable protease. We found that protease treatment removed the histidine tag but that the ATM protein remained intact (Fig. 3*C*). When detergent was added to solubilize the phospholipid bilayer, protease K was able to access and digest Atm1. These results suggest that the NBDs of both ATM3 and Atm1 are exposed to the exterior of the membrane vesicles and therefore that the transporter is in the physiological orientation in the plasma membrane of *L. lactis*.

**FIGURE 4.**
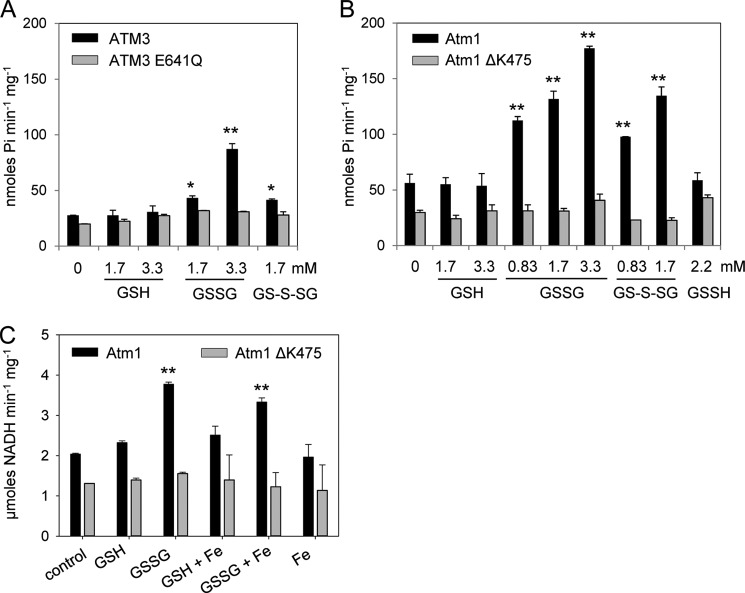
**ATPase hydrolysis measurements of ATM3 and Atm1.** *A* and *B,*ATP hydrolysis rates of purified *Arabidopsis* ATM3 (*A*) and yeast Atm1 (*B*) with GSH, GSSG, GS-S-SG (containing GS-S*_n_*-SG and GSSG in a 1:2 molar ratio) and GSSH (containing GSSH and GSSG in a 1:1 molar ratio) at the indicated concentrations. The mutant proteins ATM3 E641Q and Atm1ΔK475, which cannot hydrolyze ATP, were used as controls. The release of phosphate (P_i_) was measured by colorimetric detection using malachite green. The results are representative of two independent experiments (*A,* mean ± S.D.) or the average ± S.E. of three independent experiments (*B*). *, < 0.05; **, < 0.01 (ANOVA and post hoc Tukey's test). *C,* ATP hydrolysis rates of purified yeast Atm1 in the presence of 1.7 mm GSH or 1.7 mm GSSG and 0.1 μm FeCl_2_ (*Fe*). The release of ADP was measured using the NADH-coupled method because iron interfered with the malachite green method. The mutant Atm1 ΔK475 was used as control. The data shown are representative of two experiments (mean ± S.D.), **, *p* < 0.01 (ANOVA and post hoc Tukey's test).

Both ATM3 and Atm1 were purified to homogeneity using Ni-NTA affinity chromatography (Fig. 3*D*) and retained their ATPase activity in detergent solution (Fig. 4, *A* and *B*). The purified yeast Atm1 had a higher basal ATPase activity than *Arabidopsis* ATM3 as measured by colorimetric detection of P_i_ release from ATP by malachite green. As expected, the E641Q mutation in ATM3 and the ΔK475 deletion in Atm1 led to a substantial decrease in the observed ATPase activity (Fig. 4, *A* and *B*) confirming that the measured activity was due to the expressed ATM proteins.

##### ATPase Activity of ATM Transporters Is Stimulated by GSSG but Not GSH

Stimulation of ATPase activity of ABC transporters by transported substrates is well documented for a wide variety of these proteins (44, 45). Therefore, ATP hydrolysis rates of ATM3 and Atm1 were measured in the presence of a range of potential substrates, such as GSH, GSSG, thiols, sulfur compounds, and glutathione conjugates. Glutathione persulfide (GSSH) was produced chemically by mixing GSSG and Na_2_S. The efficiency of the reaction was analyzed by quantification of sulfane sulfur (S^0^) using the cold cyanolysis method, a colorimetric assay based on the formation of a ferric thiocyanate complex (46), which showed that 33% of the GSSG was converted to GSSH, yielding a 1:1 molar ratio of GSSH and GSSG. A GSSG polysulfide mixture was generated from elemental sulfur (S_8_) and GSH as described previously (36). Mass spectrometry revealed the presence of GS-S*_n_*-SG (*n* ≤5) and GSSG at a molar ratio of ∼1:2 and no other significant reaction intermediates (data not shown).

Neither ATM3 nor Atm1 showed a significant stimulation of ATPase activity by GSH, GSSH, cysteine, acetylcysteine, Cys-Gly, dithiothreitol, or other compounds with free thiols (Fig. 4, *A* and *B,* and data not shown). There was also no stimulation by cystine (disulfide of cysteine), lactoylglutathione, sulfite, or sulfide. However, a significant enhancement of ATPase activity was measured in the presence of GSSG and GS-S-SG/GSSG, and this stimulation was concentration-dependent and similar for both substrates (compare 0.83 and 1.7 mm values for each substrate). By contrast, the GSSH/GSSG mixture did not stimulate ATPase activity, indicating that GSSH inhibits the stimulatory effect of GSSG. For both *Arabidopsis* ATM3 and yeast Atm1, the stimulation of P_i_ release was ∼3-fold in the presence of 3.3 mm GSSG (*p* < 0.01). This is in the same range as observed for other ABC exporters (47–49).

Next, we tested whether ferrous iron was able to stimulate ATPase activity, either alone or in combination with GSH or GSSG. Because of redox chemistry between iron and malachite green, we measured the ATPase activity in an indirect enzyme assay in which the release of ADP is coupled to NADH oxidation (35). Nevertheless, FeCl_2_ concentrations of more than 0.1 mm interfered with the reaction. We confirmed that GSSG stimulated the ATPase activity of yeast Atm1 ∼2-fold (Fig. 4*C*), but neither iron alone nor iron in combination with GSH or GSSG had a stimulatory effect.

##### ATMs Can Mediate ATP-dependent GSSG and GS-S-SG Transport

**FIGURE 5.**
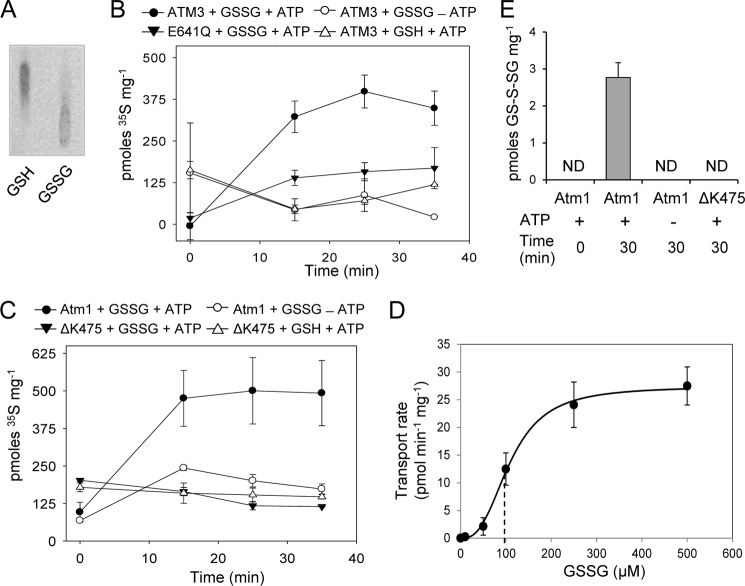
**Substrate transport measurements in inside-out membrane vesicles containing ATM3, ATM3E641Q, Atm1, or Atm1ΔK475.** *A,* separation of [^35^S]GSH and [^35^S]GSSG (0.95 μCi/mmol) by TLC. The samples contained 8 nCi of radioactivity. *B* and *C,* transport of 250 μm [^35^S]GSH or GSSG in lactococcal inside-out membrane vesicles containing *Arabidopsis* ATM3 (*B*) or yeast Atm1 (*C*). The mutant proteins with amino acid changes E641Q (in ATM3) and ΔK475 (in Atm1), which cannot hydrolyze ATP, were used as controls. Alternatively, ATP was omitted from the reaction (−*ATP*). All values are background-subtracted and represent the mean ± S.E. of three independent experiments. *D,* initial rates of Atm1-mediated transport of [^35^S]GSSG in inside-out membrane vesicles were determined at substrate concentrations between 10 and 500 μm. The data were fitted to the three-parameter Hill equation, giving an apparent average *K_m_* of 109 ± 6 μm GSSG and *V*_max_ of 27.4 ± 0.8 pmol min^−1^ mg^−1^ of membrane protein, with a Hill number of 2.8 ± 0.4. *E,* transport of GS-S-SG in inside-out membrane vesicles containing Atm1, quantified by LC-MS/MS. A GSSG/GS-S-SG mixture was added as substrate in the transport assay to a final concentration of ∼100 μm GS-S-SG and 500 μm GSSG. The value is the mean ± S.E. (*n* = 6). *ND,* not detectable.

To further assess the substrate specificity of ATM3 and Atm1, we investigated whether GSH or GSSG are transported into lactococcal inside-out membrane vesicles by rapid filtration. For this purpose, membrane vesicles containing the respective proteins were incubated with 250 μm ^35^S-labeled GSH or GSSG (0.95 Ci/mmol). The reaction mixture contained an ATP-generating system, ATP (where indicated), and MgCl_2_. GSSG was prepared by oxidation of GSH with hydrogen peroxide (see “Experimental Procedures”). To confirm the purity of the substrates, we analyzed 8 nCi on silica TLC plates (Fig. 5*A*). Clear separation of the two compounds under these conditions confirmed that the GSH sample did not contain detectable [^35^S]GSSG and that the GSSG working stock solution contained less than 20% [^35^S]GSH.

We observed ATP-dependent uptake of [^35^S]GSSG in membrane vesicles expressing ATM3 (Fig. 5*B*) or Atm1 (Fig. 5*C*). By contrast, [^35^S]GSH did not accumulate in the membrane vesicles. The accumulation of radiolabel was dependent on functional ATM protein as neither ATM3 E641Q nor Atm1 ΔK475 displayed significant transport activity. Kinetic analysis of Atm1-mediated [^35^S]GSSG transport at a substrate concentration between 10 and 500 μm revealed an apparent affinity (*K_m_*) of ∼109 ± 6 μm (Fig. 5*D*), which is in a similar range as described for mammalian MRP1 (ABCC1) (50).

To investigate whether GS-S-SG can be transported, we developed a transportomics approach (51) in which the accumulated substrates in inside-out membrane vesicles were identified by LC-MS/MS. Membrane vesicles containing Atm1 were incubated with GS-S-SG/GSSG mixture. After 30 min incubation in the presence of ATP followed by rapid filtration and washes, 3.1 ± 0.6 pmol of GS-S-SG per mg of protein was detected in Atm1-containing vesicles. In contrast, GS-S-SG was not detected in control membrane vesicles containing the ΔK475 mutant of Atm1 (Fig. 5*E*). The Atm1 vesicles also accumulated 82.8 ± 8.4 pmol of GSSG per mg of protein.

**FIGURE 6.**
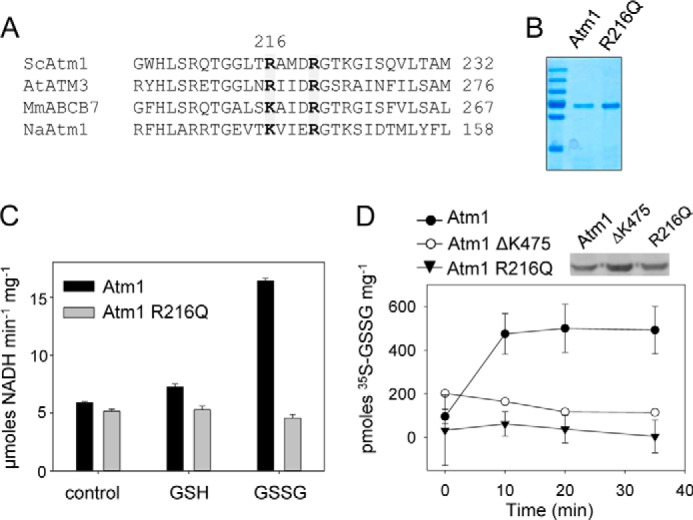
**Characterization of the arginine R216Q mutant.** *A,* alignment of Arg-216 and Arg-220 in yeast Atm1 with *Arabidopsis* ATM3, mouse ABCB7, and NaAtm1 from the α-proteobacterium *Novosphingobium aromaticivorans*. The two arginines are mostly conserved in the ATM family (marked in *gray*), although a lysine is found in ABCB7 and NaAtm1. *B,* Coomassie-stained purified proteins. *C,* stimulation of ATPase activity in purified Atm1 and the Atm1 R216Q mutant as measured using the NADH-coupled assay. Values are mean ± S.D. and representative of three independent experiments. Stimulation of Atm1 by GSSG was significant (*p* < 0.01; ANOVA and post hoc Tukey's test). *D,* ATP-dependent transport of 250 μm [^35^S]GSSG (0.95 Ci/mmol) in inside-out membrane vesicles containing the Atm1 R216Q mutant protein was compared with the activity of wild-type Atm1 and the ΔK475 mutant. Expression of Atm1 and the mutant proteins was confirmed by protein blot analysis (*inset*). The values represent the mean ± S.E. (*n* = 3).

To provide evidence for direct interactions between Atm1 and GSSG, we mutated the first arginine in a conserved (R/K)*XXX*R motif in the transmembrane domain of Atm1 (Fig. 6*A*). The R216Q change in Atm1 did not affect the stability of the protein or its basal ATPase activity (Fig. 6, *B* and *D*). Interestingly, the ATP hydrolysis rate of Atm1 R216Q could not be stimulated by GSSG (Fig. 6*C*). This correlated with the inability of the mutant protein to mediate [^35^S]GSSG transport in inside-out membrane vesicles (Fig. 6*D*), providing further evidence that GSSG transport requires functional Atm1 in our measurements.

Overall, our experimental evidence indicates that GSSG and GS-S-SG but not GSH are transported in a micromolar range of concentrations by the ATM transporters. This correlates well with the ATP hydrolysis measurements shown above, which did not show any stimulation by GSH under these conditions.

##### The atm3-1 Mutant Phenotype Is Severely Enhanced by Depletion of the Mitochondrial Sulfur Dioxygenase ETHE1

Our *in vitro* data showed that ATM3 transports GSSG and, by analogy with our transportomics results for yeast Atm1, one expects ATM3 to also transport GS-S-SG. To find evidence for sulfur transport *in vivo*, we analyzed isolated mitochondria from *atm* mutants for persulfide (S^0^) or sulfide (S^2−^), but we did not find an increase in either compound. Either our analytical methods are not sensitive enough or (per)sulfide accumulating in mutant mitochondria is rapidly detoxified.

Recently, two distinct mitochondrial activities for removal of either S^2−^ or S^0^ have been characterized in *Arabidopsis*. A mitochondrial isoform of *O*-acetylserine(thiol)lyase (EC 2.5.1.47) catalyzes the assimilation of S^2−^ by replacing the activated acetyl moiety in *O*-acetyl-l-serine to produce cysteine and acetate (24, 52). A knock-out mutant of mitochondrial *OAS-TL* (*oastlC*) has been characterized previously and displayed a mild growth defect (24). ETHE1 is a sulfur dioxygenase (EC 1.13.11.18) that oxidizes S^0^ to sulfite using GSH as a cofactor to form the intermediary substrate GSSH (25, 53, 54). Disruption of *ETHE1* caused embryo lethality (54), but a promoter mutant with strongly decreased transcript levels and sulfur dioxygenase activity is viable and displayed only a mild growth defect under normal conditions (25).

**FIGURE 7.**
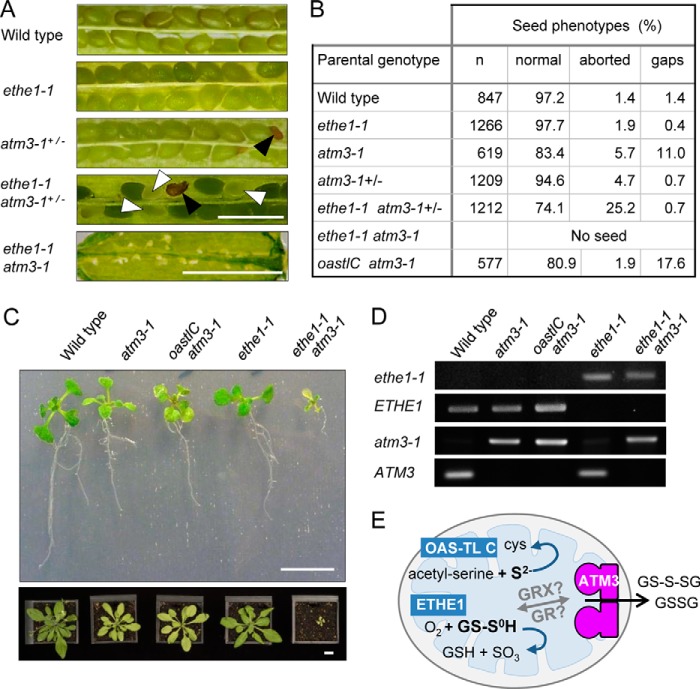
**ATM3 interacts genetically with *ETHE1* but not with *OASTL C*.** The *atm3-1* mutant was crossed with the *oastlC* knock-out mutant lacking the mitochondrial *O-*acetylserine(thiol)lyase or with the *ethe1-1* mutant, which has strongly depleted levels of the mitochondrial sulfur dioxygenase specific for GSH-persulfide as substrate. *A,* detail of opened seed pods from wild type and the indicated mutant plants. *Black arrowheads* indicate seeds aborted early in development which have turned brown; *white arrowheads* point to seeds delayed in development that will not mature. *Scale bar,* 1 mm. *B,* numbers of healthy seed, aborted seed, or nonfertilized ovules (gaps) in seed pods of the indicated lines. For each parental genotype, two separate plants were analyzed, or five plants in the case of *ethe1-1 atm3*+/−. *C,* 16-day-old seedlings with the indicated genotypes grown on agar plates (*top*) were transferred to soil and grown for another 2 weeks (*bottom panel*). *Scale bar,* 1 cm. *D,* PCR analysis to confirm the genotypes of the plants shown in *C. E,* schematic to illustrate the relation of ATM3 and the reactions catalyzed by OAS-TL C and ETHE1 in the mitochondrial matrix.

To investigate whether depletion of either OAS-TL C or ETHE1 activity would enhance the phenotype of *atm3* mutants, the *oastlC* and the *ethe1-1* mutants were crossed with *atm3-1*. We isolated the expected frequency of *oastlC atm3-1* double mutants (17:61 from an *oastlC*^+/−^ *atm3-1* parent), but we initially failed to find the double mutant of *ethe1-1* and the *atm3-1* allele. Inspection of the siliques of plants homozygous for *ethe1-1* and heterozygous for *atm3-1* revealed that about 25% of the seeds aborted (Fig. 7, *A* and *B*). Some seeds aborted early during development, whereas most died shortly before maturity. More careful scrutiny of the germinated seedlings revealed a very low percentage (1.9%) of small, pale seedlings that survived after transfer to soil (Fig. 7*C*). PCR analysis confirmed these were *ethe1-1 atm3-1* double mutants (Fig. 7*D*). The *ethe1-1 atm3-1* plants were infertile (Fig. 7*A*). In contrast, *oastlC atm3-1* mutants were similar in appearance to the *atm3-1* parental line. These data suggest that toxic persulfide, but not sulfide, accumulates in mitochondria when ATM3 is nonfunctional.

## DISCUSSION

Only a small number of ABC transporters are found in the inner mitochondrial membrane, where members of the mitochondrial carrier family are far more abundant. ATMs have been maintained during the evolution of the eukaryotic cell, apart from a few exceptions in unicellular parasitic eukaryotes, suggesting that they transport compounds essential for conserved biochemical pathways. In the light of the proposed role of ATMs in Fe-S protein biogenesis in the cytosol, and previous implication of glutathione (18, 20, 22), we focused on glutathione conjugates as possible substrates of ATMs. We found that ATP hydrolysis activity of plant ATM3 and yeast Atm1 is stimulated by GSSG but not by GSH, and that both proteins preferentially transport GSSG (Figs. 4 and 5). This substrate specificity is not unprecedented for ABC transporters and has also been reported for a bacterial Atm1-type transporter (12) and for MRP1/ABCC1 (50). In contrast, a previous study by Kunhke and co-workers (23) found that yeast Atm1, which was expressed in *E. coli* and reconstituted into liposomes, was stimulated by a range of molecules with a free thiol group, including GSH. Interestingly, their study showed that GSSG also stimulated ATPase activity but not other disulfides. The discrepancy with our study may be due to the choice of bacterial expression system. The *Lactococcus* NZ9000 strain used here does not produce glutathione; therefore, all effects seen are due to exogenously added GSH or GSSG to the *in vitro* assays.

Expression of roGFP2-GRX1 sensors showed that plant *atm3* mutants accumulate relatively more GSSG in the mitochondrial matrix (Fig. 2, *B* and *C*), providing *in vivo* support for this substrate. It should be pointed out that although the change in the mitochondrial glutathione redox state is significant, the overall effect is modest. This is not surprising considering that plant mitochondria contain an active GSH regeneration system consisting of GSSG reductase (GR2). GR2 is dual localized to the mitochondria and plastids (55). Depletion of GR2 from the mitochondria, in a *gr2* mutant expressing only plastid GR2, led to a highly oxidized mitochondrial glutathione pool.^8^ Oxidation of the mitochondrial glutathione pool can happen in the presence of GR2, for instance under stress conditions (39), although reasons for this phenomenon are not yet known. We did not observe a redox change in the cytosolic glutathione pool in *atm3* mutants. Decreased export of GSSG resulting in over-reduction of the cytosol is unlikely to be detectable using the roGFP2 reporter, which is already close to its fully reduced state under control conditions (39). In addition, the high activity and low *K_m_* value of cytosolic GR (GR1) for GSSG will efficiently buffer any variation in organellar export of oxidized glutathione.

The relatively small changes in the cellular glutathione redox state in *atm3* mutants suggest that the primary role of ATM3 is not to mediate export of GSSG from the mitochondria. Instead, we propose that GSSG serves as a vehicle to transport persulfide (sulfane sulfur with oxidation state 0), which is required for both Fe-S cluster assembly and Moco biosynthesis. The activated state of persulfide is important for the formation of Fe-S clusters, and sulfide (S^2−^) cannot be used for this purpose (4, 56). For reasons that are not yet understood, the cysteine desulfurase that generates persulfide for Fe-S cluster assembly resides in the mitochondrial matrix of plants and yeast. Export of persulfide in the form of RS-S^0^H across the inner mitochondrial membrane would be a challenge because of its reactivity. However, persulfide in the form of GS-S-SG is relatively more stable. We found that GSSH could not stimulate the ATPase activity of ATM3 and Atm1, and in fact it inhibited the stimulatory effect of GSSG. In contrast, GS-S-SG did not have an inhibitory effect on stimulation by GSSG (Fig. 4, *A* and *B*), and accumulated in inside-out membrane vesicles containing Atm1 in a reproducible manner (Fig. 5*E*). Recent advances in the synthesis and quantitative analysis of glutathione polysulfides by Ida *et al.* (57) will enable a more detailed kinetic analysis of GS-S-SG transport by ATMs in the near future.

The severely compromised growth of the *ethe1-1 atm3-1* double mutant compared with either parent suggests that an excess of glutathione persulfide accumulates in *atm3-1* mutants but is rapidly detoxified by the sulfur dioxygenase ETHE1 localized in the mitochondrial matrix (Fig. 7*E*). GS-S-SG would need to be chemically or enzymatically converted to GSSH to be a substrate of ETHE1, and GR2 or glutaredoxins are likely to catalyze this conversion (57). It should be noted that ETHE1 itself does not play a role in Fe-S cluster assembly (25), and the gene is absent from yeast. The physiological role of ETHE1 appears to be restricted to detoxification of persulfide bound to GSH rather than protein (53) and, in plants, to the catabolism of cysteine in cells undergoing high protein turnover (25).

In conclusion, we have identified two substrates of the mitochondrial ABC transporters, GSSG and GS-S-SG. Further biochemical studies are needed to establish how GS-S-SG is formed and delivered to the transporter and how the persulfide is transferred across the mitochondrial intermembrane space and delivered to cofactor assembly proteins in the cytosol.

## Footnotes

8 A. J. Meyer, unpublished data.

## Footnotes

7 The abbreviations used are:

Fe-S: iron-sulfur
ABC: ATP-binding cassette
ATM: ABC transporter of the mitochondria
BSO: buthionine sulfoximine
DDM: β-d-dodecyl maltoside
GR: glutathione disulfide reductase
GRX1: glutaredoxin 1
GSH: glutathione
GSSG: glutathione disulfide
GS-S-SG: glutathione trisulfide
LMNPG: laurylmaltoside neopentylglycol
Moco: molybdenum cofactor
MTS: mitochondrial targeting sequence
NBD: nucleotide-binding domain
NTA: nitrilotriacetic acid
roGFP: redox-sensitive GFP
ANOVA: analysis of variance.
